# The health consequences of informal employment among female workers and their children: a systematic review

**DOI:** 10.1186/s12992-023-00958-1

**Published:** 2023-08-17

**Authors:** Amanda Emma Aronsson, Pilar Vidaurre-Teixidó, Magnus Rom Jensen, Solvor Solhaug, Courtney McNamara

**Affiliations:** 1 Centre for Global Health Inequalities Research (CHAIN), Department of Sociology and Political Science, Norwegian University of Science and Technology (NTNU), Trondheim, Norway; 2 Library Section for Research Support, Data and Analysis, University Library, Norwegian University of Science and Technology (NTNU), Trondheim, Norway; 3 Population Health Sciences Institute, Newcastle University, Newcastle Upon Tyne, UK

**Keywords:** Informal employment, Health inequalities, Women’s and children’s health, Systematic review

## Abstract

**Background:**

Informal employment is unprotected and unregistered and it is often characterized by precarious working arrangements. Although being a global phenomenon and the most common type of employment worldwide, scholarly attention to its health effects has only recently accelerated. While there is still some debate, informal employment is generally understood to be detrimental to workers’ health. However, because women are more vulnerable to informality than men, attention is required to the health consequences of female workers specifically. We conducted a systematic review with the objective to examine the global evidence on the consequences of informal employment, compared to formal employment, on the health of female workers and their children.

**Methods:**

We searched peer-reviewed literature in Embase, Medline, PsychInfo, Scopus and Web of Science up until November 11, 2022. No restrictions were applied in terms of year, language or country. Individual-level quantitative studies that compared women of reproductive age in informal and formal employment, or their children (≤ 5 years), were eligible for inclusion. If studies reported outcomes per subgroup level, these were included. Study quality was assessed using the Joanna Briggs Institute checklist and a narrative synthesis of the results were conducted.

**Results:**

13 articles were included in the review, looking at breastfeeding outcomes (n = 4), child nutritional status and low birthweight (n = 4), antenatal health (n = 3), and general health outcomes for women (n = 2). The overall evidence from the included studies was that compared to formal employment, there was an association between informal employment and worse health outcomes, especially on child nutritional status and antenatal health. The evidence for breastfeeding outcomes was mixed and showed that informal employment may be both protective and damaging to health.

**Conclusion:**

This review showed that informal employment is a potential risk factor for health among female workers and their children. Further research on the pathways between informal employment and health is needed to strengthen the understanding of the health consequences of informal employment.

**Supplementary Information:**

The online version contains supplementary material available at 10.1186/s12992-023-00958-1.

## Introduction

Globally, 61% of all workers are informally employed of which about half are women who are especially vulnerable towards informality [1]. Informal work encompasses unregistered employment arrangements without employment-based social protection and other employment protections, such as sick leave, pensions or annual leave [1]. Most informal workers are, for example, street vendors, domestic workers, home-based workers or trash pickers/recyclers. Triggered by global economic processes [2], labour market transformations have resulted in a growth of non-standard employment where employment-based protections are reduced and contractual statuses are more uncertain [3], thus increasing the likelihood of workers entering informality. Therefore, while still most widespread in low- and middle-income countries, the non-standard employment type of informal employment is increasingly relevant also in high-income countries.

Employment in general, is a known health determinant [4] that is most commonly explained to influence health through working conditions such as working hours and hazardous working environments [5, 6]. The link between employment and health is also influenced by macro-level policy contexts and it intersects with individual factors such as socioeconomic status, gender, age, place of residence and race/ethnicity [5]. The interplay between these factors contributes to the potential barriers that workers may face in terms of accessing health benefiting policies or services. There is a growing body of work that has specifically examined the links between informal employment and health. A recent review of this literature found mixed results indicating that informal employment is not always associated with poorer health outcomes compared to formal employment. Instead, the health implications of informal work are found to have gendered effects that intersect with broader sociopolitical conditions and with women’s “double burden of work” where their responsibility to carry out both paid work and unpaid work, such as caring for the household and children, plays a central role [7].

On one hand, this double burden of work can mean that formal employment brings greater health benefits to women than men. Alfers and Rogan [8] found that increasing formality indeed had greater health benefits for women. This could partly be explained by the protections that formal employment offers, such as maternity leave, which can reduce some of the double burden and its consequences. On the other hand, it can mean that in some countries where social protection availability is scarce also to formal workers, there is little health benefits afforded to women working in the formal versus informal sector. Examining how the health effects of informal employment can differ by welfare state regime, Rodriguez-Loureiro et al. [9] found that in highly ‘familialist’ countries, where social security systems are weak, formally working women did not have better health than those working informally. This is, as the authors suggest, potentially because women in these countries suffer from a similar burden of care.

Women’s double burden of work can also have implications for how employment formality impacts maternal and child health. In terms of breastfeeding, a feeding practice that can both reduce childhood mortality and lower the risk of breast cancer for the mother [10], evidence on the relationship between formality of work and health is mixed. Oddo and Ickes [11] for example, examined the association between maternal employment and exclusive breastfeeding among children < 6 months of age. They found that formal employment was significantly associated with lower odds of exclusive breastfeeding in some world regions but not in others. Specifically, formally employed mothers in East Asia, the Pacific, Latin America and the Caribbean seemed to find it more challenging to breastfeed exclusively in the first months of motherhood. In this study, formal employment was also significantly associated with lower odds of continued breastfeeding at 1 year. Other authors, however, have found that informal employment among mothers associates with delayed breastfeeding initiation [12] and worse infant feeding practices more generally [13].

Thus far, there has yet to be a systematic evaluation of the health consequences of informal employment for women’s and children’s health. The objective of this review was therefore to identify and synthesize the existing evidence on the consequences of informal (compared to formal) employment on the health of women of reproductive age (15–49 years), and the health of their children (aged ≤ 5 years).

## Methods

The review was conducted in accordance with the Preferred Reporting Items for Systematic Reviews and Meta-analyses (PRISMA) guidelines and the protocol was registered with the International Prospective Register of Systematic Reviews (PROSPERO CRD42021273014).

### Eligibility criteria

The PECOS framework (population, exposure, comparison, outcome, and study design) was used to develop the eligibility criteria:

*Population.* Studies including female workers of reproductive age (15–49 years) were eligible. Children (≤ 5 years) of female workers were also included.

*Exposure.* Informal employment measured at the individual level of women of reproductive age. Studies were included if informal employment was explicitly stated as an exposure. Since informal employment is not always explicitly identified, studies that described informal employment, without explicitly referring to it as such, were also included. For example, we included studies that examined employment characterized by no contract, oral contract, non-registered work/workers (for employees), non-registered enterprises (for self-employed and employers) or no income taxation. We included studies of informally employed employees, employers or self-employed and female workers from all occupations (for example, domestic workers, street vendors, trash pickers, home-based workers). Because both precarious workers and informal workers may lack social protection in general, this factor was not used to identify informal employment.

We excluded studies that did not identify the employment conditions as informal or did not describe informal working conditions as indicated above. Studies that described the sector as informal rather than the job or the employment conditions were also excluded since working in the informal sector can take place under formal employment conditions.

*Comparator.* Studies that compared women in informal employment with women in formal employment were included. Unless explicitly described as formal employment, studies were also included if they described employment as sufficiently formal. We took formal employment to be characterized by, for example, a written contract, tax-based income or registered work/workers. Studies that merely described an aspect of the employment, such as full-time employment or paid work, were excluded since these characteristics can be associated with both formal and informal employment.

*Outcome*. We included studies of either women’s health outcomes or children’s health. Our inclusion criteria were broad to cover any considered health outcome indicators including physical health, mental health, occupational health, maternal health and child health. Because maternal health and child health are commonly measured through health indicators such as breastfeeding outcomes and maternal health care access, these indicators were also included as they are closely related to health outcomes such as nutritional status or maternal- and child mortality. Broader health indicators outside of physical health, mental health and outcomes related to health access, such as health literacy and health awareness were excluded.

*Study design.* Quantitative, peer-reviewed studies which assessed individual level data were included. Qualitative studies, reviews and commentaries, for example, were excluded.

Studies of all languages and countries were included and no restriction in terms of publication year was applied.

### Search strategy and screening

The initial literature search took place August 13, 2021, with an updated search November 11, 2022, using the following databases: Embase (Ovid), Medline (Ovid), PsycInfo (Ovid), Scopus and Web of Science. The search string was revised, tested and applied by two research librarians (SS and MRJ) and optimized for each database and their syntax (supplementary material 1). Endnote 20 was used for the removal of duplicate references. A hand search was conducted by reaching out to experts in the field and by screening the reference lists of the included articles.

Title and abstract screening were done in blind duplicates by four reviewers (SS, MRJ, AA and PVT) using Rayyan. Any discrepancies were resolved by consensus. Departing from the AMSTAR 2 checklist for assessing the quality of systematic reviews [14], an 80% agreement rate between two reviewers of a sample of the articles to be assessed for full-text eligibility is required to allow one reviewer to continue the full-text screening. Two reviewers (AA and PVT) screened a sample (n = 50) of the studies (n = 223) with an agreement rate of 92%, which allowed one reviewer (AA) to continue with the screening of the remainder of the articles.

**Fig. 1:**
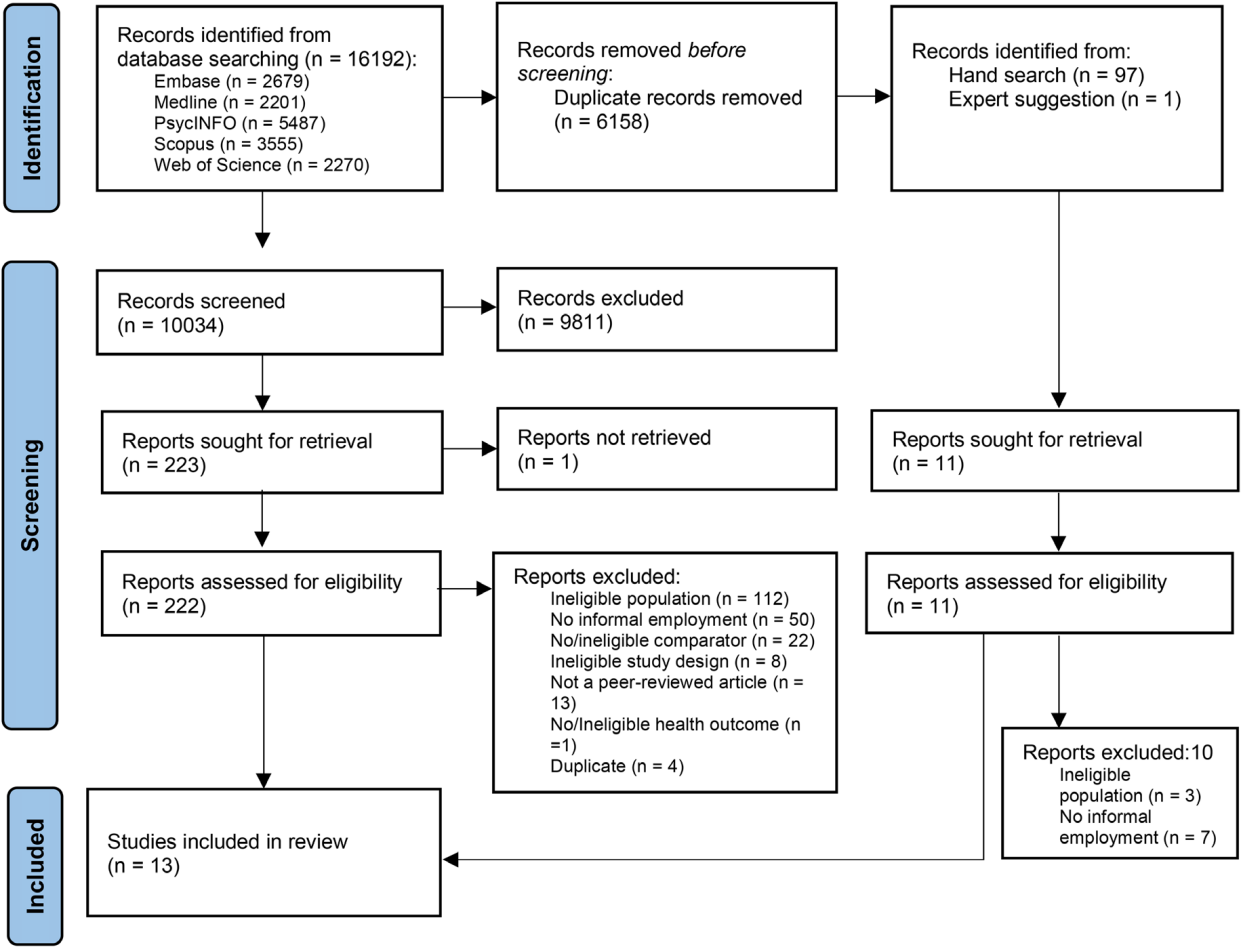
PRISMA flowchart

### Data extraction and synthesis

The data extraction was conducted by the lead author (AA) with a 10% check conducted by CM. Data on country setting, year of data collection, aim of study, study design, study population, health outcome, statistical analysis and findings related to informal employment and health was extracted into a table. Results from any subgroup according to the PROGRESS plus framework [15] was also extracted. A narrative synthesis of the results was structured by health outcomes and interpreted with consideration to larger policy contexts. No meta-analysis was done due to a heterogeneity of the study designs, the sample population and the outcomes.

The quality of the included studies was evaluated using the Joanna Briggs Institute (JBI) checklist for cross-sectional studies (supplementary material 2). The assessment was done by the lead author (AA) with a 10% check conducted by CM. The JBI checklist provides eight questions relating to the study design, measurement of variables and the statistical analysis. Every ‘yes’ was assigned one point. The quality of a paper was scored as ‘high’ if it scored between 70–100%, it was scored as ‘moderate’ between 50–69% and a result of ≤ 49% was scored as low quality. No papers were excluded based on the score and the scores were only meant to give a broad overview of the quality of individual studies as well as to provide a picture of the overall quality.

## Results

The search identified 16,192 articles, of which 10,034 remained after removing duplicates. An additional 97 articles were identified by hand searching the references of including articles. After screening the titles and abstracts, 234 articles were sought for full-text reading, of which 233 were obtained and 13 studies met the eligibility criteria (Fig. 1).

### Study characteristics

An overview of the studies’ characteristics and results are found in Table 1. Most of the 13 studies were conducted in Asia (N = 6), followed by Africa (N = 3) and South America (N = 3). One study was conducted in Europe. The papers were published between 1991 and 2022 with about half (N = 7) being published since 2015. The sample sizes varied from 64 to 3878 participants and most of the papers (N = 8) had a sample size smaller than 1000. All studies were cross-sectional. The aims of the papers varied. About half (N = 7) explicitly aimed to test the effect of employment on an outcome, while the other half (N = 6) tested a range of factors associated with a health outcome of which employment was one of those factors or it was used as a control variable. The health outcomes varied across papers and related to breastfeeding (N = 4), child nutritional status (N = 3), low birth weight (N = 1), antenatal care (N = 3) and general adult health outcomes (N = 2). Authors often used multiple indicators to measure breastfeeding outcomes and nutritional status, resulting in a total of 17 unique health outcome indicators being used across the studies. Most outcomes were measured through self-report (N = 9), three papers used objective measures and one paper was unclear in its measuring methods. Two papers reported subgroup results and specifically examined differences in effects between age groups and the number of years immigrants had lived in the new country.

**Table 1:** Description of included studies

Author (year)	Country	Study design	Sample (N)	Outcome	Statistical analysis	Main finding of employment and health	Quality
*Breastfeeding*							
Hao et al., (2022)	China	Cross sectional	Mothers aged 20–42 with children under the age of 2 (N = 1677)	Breastfeeding outcome: Exclusive breastfeeding 0–6 months	Logistic regression	Women in informal employment had lower odds of practicing exclusive breastfeeding compared to mothers in formal employment (**OR = 0.45, 95% CI = 0.28–0.72, p = 0.004)**	Moderate
Sanches et al., (2011)	Brazil	Cross sectional	Mother/infant pairs of infants with low birth weight (N = 170)	Breastfeeding outcome: Interruption of exclusive breastfeeding up to three months	Hierarchical poisson multiple regression	Informal employment was found to be a protective factor to interruption of exclusive breastfeeding (**PR = 0.70, 95% CI = 0.55–0.89, p = 0.003**)	High
Nkrumah (2016)	Ghana	Cross sectional	Mother/infant pairs (children aged 0–7 months) (N = 225)	Breastfeeding outcomes: Exclusive breastfeeding (0–6 months) and breastfeeding frequency (≥ 8 times/day or < 8 times/day)	Chi square	84% of women in informal employment exclusively breastfed compared to 16% of women in formal employment. 91% of women in informal employment breastfed more frequently, while 9% of women in formal employment breastfed more frequently. The associations between mothers in formal and informal employment and breastfeeding outcomes were significant (***p*** **= 0.020** for exclusive breastfeeding and ***p*** **= 0.021** for breastfeeding frequency)	Moderate
Chen et al., (2019)	China	Cross-sectional	Mothers with children under 12 months (N = 3878)	Breastfeeding outcomes: Early initiation of breastfeeding (EIB), exclusive breastfeeding under six months (EBF), predominant breastfeeding under six months (PBF), children ever breastfed (ever BF), current breastfeeding (CBF)	Logistic regression	Both migrants **(AOR = 0.69 CI = 0.51–0.92**) and locals **(AOR = 0.71, CI = 0.54–0.94)** that were informally employed had significantly lower odds to CBF compared to those who were formally employed. Informally employed migrants also had significantly lower odds to EIB as compared to formally employed migrants **(AOR = 0.59, CI = 0.38–0.90).** There was no difference in EIB between local informally employed or local formally employed women (AOR = 0.93, CI = 0.62–1.39). There was no difference between employment types and any other breastfeeding practices (Ever BF, EBF or PBF)	High
***Child nutritional status and low birthweight***							
Jafree et al., (2015)	Pakistan	Cross sectional	Employed women in paid work with at least one child born in the last 5 years (N = 2515)	Low birthweight	Multivariate binary logistic regression	Children of mothers in informal employment had lower odds of having low birth weight, the results were not significant (AOR = 0.72, 95% CI = 0.47–1.09, p = 0.126)	High
Engle (1991)	Guatemala	Cross sectional	Mother/child pairs (children aged 8–35 months old) (N = 239)	Nutrtional status (anthropometric measurements): Height-for-age z score (HAZ), weight-for-age z score (WAZ) and weight-for-height z scores	ANCOVA	HAZ: the mean was − 1.79 for children of mothers in informal work and − 1.66 for children of mothers in formal work WAZ: the mean was − 1.39 for children of mothers in informal work and − 1.08 for children of mothers in formal work Weight for height: the mean was − 0.34 for children of mothers in informal work and − 0.05 for children of mothers in formal work In the initial ANOVA, there was a significant relationship between the mother’s type of work with height for age and weight for age, the relationship was no longer significant after adjusting for confounders. There was no association between mothers’ type of work and weight for height	High
Toyama (2001)	Indonesia	Cross sectional	Children under the age of 5 (N = 64)	Nutrtional status: Height-for-age z score (HAZ) and weight-for-age z score (WAZ)	Mulitple linear regression	Children of mothers in informal work were significantly more likely to have poor nutritional status compared to children of mothers in formal work. The mean HAZ for children of informally employed mothers was − 1.56 and mean HAZ was − 0.14 for children of formally employed mothers was **(*p*** **= < 0.01**). Mean WAZ for children of informal mothers was − 1.75 compared to a mean of -0.67 among children of formally employed mothers (***p*** **= < 0.01**)	Moderate
Nakahara et al., (2006)	Nepal	Cross sectional	Mother/child pairs (children aged 10–24 months) (N = 72)	Nutritional status (anthropometric measurements): Underweight (weight-for-age z score ≤ -2) and stunting (height-for-age z score ≤ -2)	Logistic regression	Underweight: Children of mothers in informal employment had higher odds of being underweight compared to children of mothers in formal employment **(AOR = 31.07; 95% CI = 1.46–663; p = 0.03).** Stunting: Children of mothers in informal employment had higher odds of reporting stunting compared to children of mothers in formal employment but these results were non-significant (AOR = 1.61; 95% CI = 0.17–15.5; p = 0.68)	High
***Antenatal care***							
Ha et al., (2015)	Vietnam	Cross sectional	Mothers who gave birth in the last year, living in rural areas of Vietnam (N = 907)	Utilization of more than four antenatal care services (ANC4+) services	Multivariate logistic regression	Mothers with a formal job, i.e., business owners **(OR = 3.1, 95% CI = 1.08–8.78)** and government officials **(OR = 1.9, 95% CI = 1.11–3.26)**, had higher odds of using ANC4 + services than mothers with informal jobs	High
Ihomba et al., (2020)	Kenya	Cross sectional	Women admitted to a local referral hospital in Kenya with pregnancy or childbirth related complications (N = 353)	Birth preparedness and complication readiness (BPCR)	Chi square	Formal employment was associated with women having higher odds of reporting BPCR **(OR = 4.14, 95% CI = 2.51–6.82, p < 0.001**) compared to women in informal employment	High
Agbozo et al., (2022)	Ghana	Cross sectional	Pregnant women attending antenatal clinics (N = 817)	Adherence to appointments for Gestational Diabetes Mellitus (GDM) testing	Logistic regression	Women in formal employment had higher odds of adhering to their scheduled GDM test compared to informal workers, the result was not significant (AOR = 1.46, 95% CI = 0.56–3.77, p = 0.430)	High
***General health outcomes***							
Santana and Loomis (2004)	Brazil	Cross sectional	Paid workers aged 18–65, stratified by age and sex (N = 1370)	Non-fatal occupational injuries	Poisson regression	There was no significant association between informal employment and non-fatal occupational injuries for any of the age groups. Workers in informal employment aged 18–21 had the highest incidence rates of occupational injuries (IRR 1.72, 95% CI = 0.33–8.83), followed by women aged 22–40 (IRR, 1.69, 95% CI = 0.90–3.16)	Moderate
Sousa et al. (2010)	Spain	Cross sectional	Foreign-born and Spanish-born workers, stratified by sex and residence status (N = 2358)	Self-rated health and mental health	Logistic regression	**Poor self-rated health**: Compared with Spanish-born formal workers Foreign-born informal workers living in Spain > 3 years had higher odds of reporting poor self-rated health (**AOR = 4.63, 95% CI = 1.95–10.97**) Foreign-born informal workers who had lived in Spain ≤ 3 had lower odds of reporting poor self-rated health (AOR = 0.53, 95% CI = 0.62–4.59), and Spanish-born informal workers had higher odds of reporting poor mental health (AOR = 1.32, 95% CI = 0.41–4.27). Neither of these association were significant **Poor mental health**: Compared with Spanish-born formal workers In the univariate model, foreign-born informal workers living in Spain > 3 years had significantly higher odds of reporting poor mental health **(OR = 2.41, 95% CI = 1.22–4.75)**. After testing for confounders, the results were no longer significant (AOR = 1.93, 95% CI = 0.95–392) Spanish-born informal workers (AOR = 1.11, 95% CI = 0.50–3.48) had higher odds of reporting poor mental health. Foreign-born informal workers living in Spain ≤ 3 had lower odds of reporting poor mental health (AOR = 0.71, 95% CI = 0.20–2.50). Neither of these associations were significant	High

Of the 13 papers, eight were rated as high quality, three of moderate quality and two as low quality. The domain most often identified as a risk of bias was whether the outcome measure was measured in a valid and reliable way and many papers scored low in this domain because they used self-reported outcomes. Many studies also did not provide sufficient information on confounding variables nor any strategies to deal with confounding variables.

### Breastfeeding outcomes

Four papers examined self-reported breastfeeding outcomes, with mixed results. Two studies found that informal employment was protective of breastfeeding outcomes. Specifically, Sanches et al. [16] aimed to identify factors associated with interruption of exclusive breastfeeding at the age of three months among a sample of mothers to infants born with low birth weight. The study, conducted in Brazil, showed that informally employed mothers had a lower prevalence of interruption of exclusive breastfeeding than those in formal employment [16]. Similarly, Nkrumah [17], in examining the effect of work and employment factors on exclusive breastfeeding and breastfeeding frequency in Ghana, found that informal employment was associated with better breastfeeding outcomes.

The two papers that found informal employment to be associated with worse breastfeeding outcomes were both conducted in China. Hao et al. [18] aimed to identify factors associated with exclusive breastfeeding and here, informal employment was associated with lower odds of a mother exclusively breastfeeding. The other study [19] investigated the association between employment type and five different breastfeeding outcomes among migrants and locals. For the outcome of currently breastfeeding they found that both migrant and local informally employed mothers had lower odds of currently breastfeeding. They found one other statistically significant association which was that informally employed migrants had lower odds of initiating breastfeeding early. No statistically significant associations were found between informal employment and the remaining three outcome indicators, but the odds of practicing exclusive breastfeeding and predominant breastfeeding were higher among informally employed mothers and the odds of children ever being breastfed were lower.

### Child nutritional status and low birth weight

Four papers examined child nutritional status or low birthweight. Three of the papers had an explicit aim to examine the association between employment and health outcomes [20–22] while the remaining paper only controlled for employment to identify determinants of child nutritional status [23]. Of these papers, three examined nutritional status outcomes using anthropometric measurements [21–23] and one paper used self-reported birth weight [20]. Informal employment was associated with poor nutritional status among children in two papers. Toyama et al. [22] examined the association between maternal employment and weight-for-age z score (WAZ) and height-for-age z score (HAZ) among a sample of mothers and children in Indonesia. Children of mothers in informal employment had both lower HAZ and WAZ compared to children of mothers in formal employment. Similarly, a study in Nepal by Nakahara et al. [23] identified a statistically significant association between maternal informal employment and higher odds of children being underweight. Although the odds of children being stunted were also higher among children of informally employed mothers, the results were not statistically significant.

The remaining two papers found no association between informal employment and child nutritional status or low birthweight. Although in one of the studies [21], maternal informal employment in Guatemala was initially associated with lower HAZ among children aged 8–35 months. The statistical significance of the results however, disappeared after controlling for education and income. In this paper, WAZ and height-for-weight were also lower among children of informally employed mothers, but the results were not statistically significant. Finally, Jafree et al. [20] aimed to identify the association between maternal employment and low birthweight in Pakistan. Here, informal employment was associated with lower odds of low birth weight, but the result was not statistically significant.

### Antenatal care

Three papers explored outcomes related to antenatal care. Of these, two found that informal employment was associated with worse outcomes. In examining the rate of utilization of, and factors associated with the use of four or more antenatal care (ANC4+) services among pregnant women in rural areas of Vietnam, informally employed women had lower odds of using such services as compared to those in formal employment [24]. Likewise, in a sample of Kenyan mothers admitted to hospital with pregnancy- or childbirth-related conditions, Ihomba et al. [25] found that informally employed women had lower odds of reporting birth preparedness and complication readiness (BPCR), an outcome which measures plans for birth and anticipated actions in case of complications and which has been shown to reduce neonatal and maternal mortality. The third and final study aimed to identify the factors associated with adherence to scheduled Gestational Diabetes Mellitus (GDM) testing in a sample of women attending antenatal clinics in Ghana, and although the odds of adhering to scheduled appointments were lower among informally employed women, these results were not statistically significant [26].

### General health outcomes

Two studies reported on the effect of employment conditions on health outcomes for female workers. Examined outcomes were non-fatal occupational injuries in Brazil [27] and self-rated health and mental health in Spain [28].

With regards to non-fatal occupational injuries, incidence rates were found to be higher among informally employed Brazilian women and across all age-subgroups [27]. There was a stepwise increase in incidence rates for each older sub-group, although only two age groups were eligible for this review. However, neither of these results were statistically significant.

In terms of self-rated health in Spain, Sousa et al. [28] found that foreign-born informally employed migrants who had been living in Spain for three years or more, had higher odds of reporting poor self-rated health. Spanish-born informal workers were also found to have higher odds of reporting poor self-rated health while lower odds were found among foreign-born informally employed migrants who had lived in Spain for less than three years. Neither of these latter two results, however, were statistically significant.

In terms of mental health in Spain, informal employment was initially associated with worse outcomes for foreign-born informal workers living in Spain for more than three years, but after controlling for confounding factors (age, education, sector of economic activity and income), the association was no longer significant. Mirroring the findings for self-rated health above, informally employed Spanish-born workers were found to have higher odds of reporting poor mental health while foreign-born migrants who had lived in Spain for less than three years were found to have lower odds of poor mental health [28]. Again, however, neither of these latter two findings were statistically significant.

## Discussion

The objective of this review was to identify and synthesize current evidence on the association between informal (versus formal) employment and the health of women of reproductive age, along with the health of their children (aged ≤ 5 years). A total of 13 studies were included and overall, our results suggest a negative association between informal employment and women’s and children’s health outcomes. While some non-significant results were found, the majority indicated a negative association between informal employment and health. However, for outcomes related to breastfeeding, and in line with previous research [11, 12], results were mixed.

In terms of breastfeeding, the mixed evidence might be explained by the institutional contexts of the countries where the studies took place [29]. For example, paid maternity leave, work schedule flexibility and workplace facilities for breastfeeding or pumping, have been identified as key social and workplace related factors which can facilitate exclusive breastfeeding and other breastfeeding outcomes among working women in LMICs [30] and specifically in regions and countries where studies in this review were based, such as Latin America and the Caribbean [31], Ghana [32, 33] and China [34]. The two studies in our review that found formal employment to be protective of breastfeeding were undertaken in China where formal workers are legally entitled to paid maternity leave and where there are often workplace arrangements in place that allow for breastfeeding or pumping such as nursing rooms and flexible schedules [18, 19]. These contextual factors can be contrasted with those of the two studies undertaken in Brazil and Ghana that found informal employment to be associated with better breastfeeding outcomes. While workers in the formal sector, both in Brazil and Ghana, have access to paid maternity leave, there is a deficit of workplace facilities and flexible working conditions [16, 17]. In these countries then, the flexibility of informal employment in terms of working schedules might explain why informally employed mothers have better breastfeeding outcomes.

That the flexibility of informal employment was evidently not sufficient for supporting breastfeeding among informally working mothers in China suggests that flexible work is a necessary but perhaps not sufficient factor for supporting breastfeeding. A scarcity of public nursing rooms has been reported as a key reason why mothers in China choose to interrupt breastfeeding and to instead offer milk substitution [35]. Because informal work often takes place in public spaces [36], this might also explain some of the different findings between the relative protectiveness of informal work in Brazil and Ghana compared to China. Mothers in China who are not comfortable breastfeeding or pumping openly might instead choose to give milk substitutes, thus lowering the rates of exclusive- and continued -breastfeeding among informally employed mothers.

For child nutritional status, we found that informal employment had an overall negative effect and that it was specifically associated with higher odds of being underweight and stunting. This might, in part, be explained by maternal access to health care, including antenatal care which has been identified as a strong predictor of childhood stunting [37]. This idea is supported by studies in our review which found an association between informal employment and reduced odds of attending antenatal services [24] and birth preparedness and complication readiness [25]. Socioeconomic barriers are likely to explain some part of reduced access to antenatal services and BPCR among informally employed women who typically have lower pay and less access to income support policies that could compensate for lost income in case of taking time of work to attend healthcare appointments [1, 24, 38]. Other barriers to antenatal services may be related to place of residence as informal workers are more likely to live in rural areas [1] where women often face additional difficulties accessing health care facilities [38].

Finally, we found suggestive results that informal employment may have a differential health effect among workers according to migrant status and that years of residence in the new country influences this relationship. As discussed by the authors, this could potentially be explained by the healthy migrant effect [28], whereby those who recently migrated tend to have better health compared to other migrants and the general population. The interplay of factors explaining why informality has a differential effect for migrants, both compared to local workers as well as compared to within-migrant groups, needs to be further explored by taking into consideration other individual-level factors that might influence the relationship. Moreover, this supports the idea of using an intersectional lens when exploring the relationship between employment-related variables, such as informal employment, and health [5, 39].

Overall, the mixed findings across the studies in this review suggest that the consequences of informal employment on women’s and children’s health is to a large extent determined by context-specific factors both at the macro and individual level.

### Limitations

Despite our best efforts to ensure quality and comprehensiveness, these findings should be interpreted considering some limitations. First, we found only a limited number of quantitative studies. This may be because data on informal employment is difficult to collect. It would thus be useful for future work to synthesize qualitative evidence in this area. We also cannot exclude the risk of publication bias against studies reporting negative findings. This could be addressed in future work through an examination of grey literature and government reports which may be more likely to publish negative findings. Several limitations also arose at the individual study level. An important one is that all studies were cross-sectional, limiting our understanding of the causal nature of the relationship between informal work and women’s and children’s health. For example, poor health outcomes (either among the mother or her child) might lead a woman to seek informal employment. Further, cross-sectional designs do not capture trends over time which conceivably could affect the strength of the relationship between informal work and health. Crucially, many of the included studies had low sample sizes which reduces the statistical power of the results, this can increase the likelihood of type II errors, that is, the risk of rejecting the alternative hypothesis despite it being true. A strength in the design of the review lies in its broad inclusion criteria where studies from all countries, of all years and in all languages were included, thus increasing the likelihood of identifying all relevant articles and contributing to a comprehensive synthesis of existing quantitative evidence.

## Conclusion

Our review provides the first systematic look at the relationship between informal employment and women’s and children’s health. Our findings suggest that informal employment is damaging to the health of female workers of reproductive age, and to the health of their children, particularly in terms of antenatal care utilization and child nutritional status. Consistent with previous work, however, results were mixed, and notably with regards to breastfeeding outcomes. This is likely due to institutional factors related to paid maternity leave, workplace facilities and flexible working conditions. In policy debates about how to protect informally employed women and their children it should be important to remember the importance of socioeconomic resources and their access to antenatal care. Future work aiming to understand the relationship between informal employment and women’s and children’s health should focus on the moderating role of country-based policy contexts and specifically that of social protection policies like paid maternity leave, as well as employment- and workplace factors.

### Electronic supplementary material

Below is the link to the electronic supplementary material.

**Supplementary Material 1**. Example of the search strategy

**Supplementary Material 2**. Joanna Briggs Institute Checklist for analytical cross sectional studies

## Data Availability

The datasets are available through the corresponding author upon reasonable request.

## Abbreviations

PRISMA: Preferred Reporting Items for Systematic Reviews and Meta-analyses
PECO: Population, Exposure, Comparison, Outcome and Study design
WAZ: Weight-for-Age z score
HAZ: Height-for-Age z score
ANC4+: Four or more Antenatal Care Service Use
BPCR: Birth Preparedness and Complication Readiness
GDM: Gestational Diabetes Mellitus
CBF: Current Breastfeeding
EIB: Early Initiation of Breastfeeding
EBF: Exclusive Breastfeeding
PBF: Predominant Breastfeeding
Ever BF: Ever Breastfeed
