# Correction: The health consequences of informal employment among female workers and their children: a systematic review

**DOI:** 10.1186/s12992-023-00967-0

**Published:** 2023-09-01

**Authors:** Amanda Emma Aronsson, Pilar Vidaurre-Teixidó, Magnus Rom Jensen, Solvor Solhaug, Courtney McNamara

**Affiliations:** 1 Centre for Global Health Inequalities Research (CHAIN), Department of Sociology and Political Science, Norwegian University of Science and Technology (NTNU), Trondheim, Norway; 2 Library Section for Research Support, Data and Analysis, University Library, Norwegian University of Science and Technology (NTNU), Trondheim, Norway; 3 Population Health Sciences Institute, Newcastle University, Newcastle Upon Tyne, UK

Globalization and Health (2023) 19:59

10.1186/s12992-023-00958-1

Following publication of the original article, it came to the authors’ attention that there was an error in the affiliations information: the author Courtney McNamara had been assigned affiliations 2 and 3 instead of affiliations 1 and 3. The published article has since been corrected. The authors thank you for reading this erratum and apologize for any inconvenience caused.

