# Supplementary material for: The health consequences of informal employment among female workers and their children: a systematic review
Source: Global Health. 2023 Aug 17;19:59. doi: 10.1186/s12992-023-00958-1 (PMC10436452; doi:10.1186/s12992-023-00958-1)
Supplement: Supplementary file 1 — Supplementary Material 1. Example of the search strategy [file 12992_2023_958_MOESM1_ESM.docx]

**Example, search strategy (Scopus)**

TITLE-ABS-KEY ( ( wom?n OR female* OR mother* OR maternal ) OR ( child* OR toddler* OR neonat* OR baby OR babies OR newborn OR infancy OR infant*) AND ( "employment condition*" OR "informal employ*" OR "informal work*" OR "informal sector" OR "informal job*" OR "informal economy" OR "informally employed" OR "formal employ*" OR "formal work*" OR "formal sector" OR "formal job*" OR "formally employed" OR "unregistered work*" OR "informal trade*" OR "street vend*" OR "waste recycl*" OR "informal recycl*" OR "recycling work*" OR "waste pick*" OR "trash pick*" OR ragpick* OR "rag pick*" OR "street hawk*" OR "home based work*" OR "domestic work*" OR "informal min*" OR "artisanal min*" ) AND ( health OR well-being OR wellbeing OR morbidity OR mortality OR death OR illness* OR disease* OR disorder* OR injur* OR pain* OR accident* OR healthcare OR infection* OR depress* OR stress OR anxiety OR trauma OR obes* OR overweight OR malnutrition OR nutrition OR underweight OR "skin irritation" OR "hearing loss" OR hypertension OR cardio* OR violence OR abuse OR cancer OR prenatal OR postnatal OR perinatal OR pregnancy OR antenatal OR obstetric OR stunting OR wasting OR breastfeed* OR breastfed OR vaccin* OR immunization OR neurodevelopment* OR "low birth weight" OR "low-birthweight" OR "preterm" OR "pre term" OR premature* ) )
